# Supplementary material for: Resilience of Emiliania huxleyi to future changes in subantarctic waters
Source: PLoS One. 2023 Nov 2;18(11):e0284415. doi: 10.1371/journal.pone.0284415 (PMC10621989; doi:10.1371/journal.pone.0284415)

**S1 Fig. Scanning electron micrographs of Emiliania huxleyi (P1406 E. hux #1) grown at 2015 temperature and pH.** Cells were prepared for microscopy by filtration onto 0.4 µm polycarbonate filters (Whatman). The filters were allowed to air-dry before sputter-coating with gold-palladium and examination with a JEOL 6700 SEM Field emission scanning electron microscope (JEOL Ltd, Tokyo, Japan).


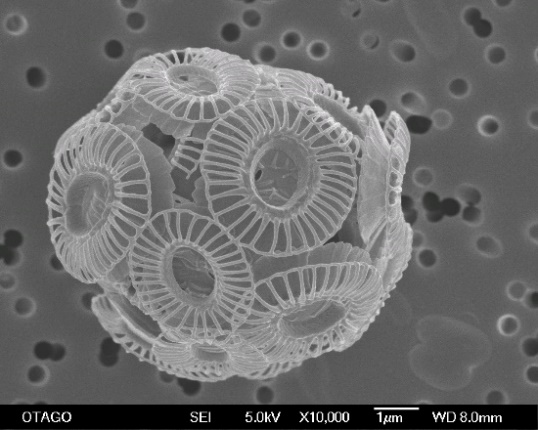

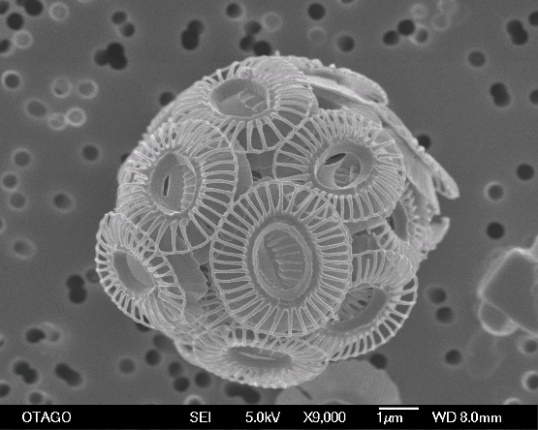

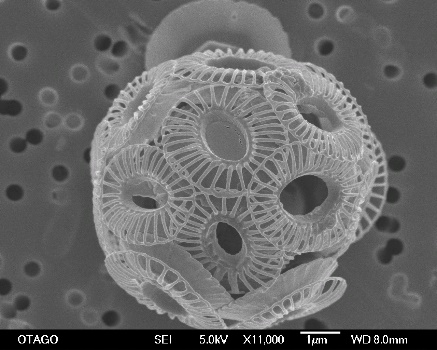

Supplement: S1 Fig — (DOCX) [file pone.0284415.s001.docx]
