# Supplementary material for: Resilience of Emiliania huxleyi to future changes in subantarctic waters
Source: PLoS One. 2023 Nov 2;18(11):e0284415. doi: 10.1371/journal.pone.0284415 (PMC10621989; doi:10.1371/journal.pone.0284415)
Supplement: S2 Fig — (DOCX) [file pone.0284415.s002.docx]

**S2 Fig. *E. huxleyi* cell contents and ratios at D670 for each treatment.** Mean presented for all parameters, n = 3, error bars indicate SEM. A. chl-a content (pg chl-a cell^-1^); B. PON content (pg PON cell^-1^); C. POP content (pg POP cell^-1^); D. POC : Chl *a* (µg ml^-1^ : µg ml^-1^); E. POC : PON (µmol L^-1^ : µmol L^-1^); F. POC : POP (µmol L^-1^ : µmol L^-1^); G. PON : POP (µmol L^-1^ : µmol L^-1^) . Significant differences indicated by b for N and N in F, c for F and F in N.

A

D

E

b

B

F

C

G

c
