# Supplementary material for: Resilience of Emiliania huxleyi to future changes in subantarctic waters
Source: PLoS One. 2023 Nov 2;18(11):e0284415. doi: 10.1371/journal.pone.0284415 (PMC10621989; doi:10.1371/journal.pone.0284415)
Supplement: S3 Fig — (DOCX) [file pone.0284415.s003.docx]

**S3 Fig. *E. huxleyi* volumetric contents and ratios and rates at D670 for each treatment.** Mean presented for all parameters, n = 3, error bars indicate SEM. A. Chl-a content (pg chl-a µm^-3^); B. PON content (pg PON µm^-3^); C. POP content (pg POP µm^-3^); D. POC content (pg POC µm^-3^); E. PIC content (pg PIC µm^-3^); F. POC uptake (pg µm^-3^ hr^-1^); G. Ca uptake (pg µm^-3^ hr^-1^). Significant differences indicated by a for N and F, by b for N and N in F, and by c for F and F in N.

a

a

a,b,c,d,e

a,b,c

A

B

C

D

E

F

G
