# Supplementary material for: Resilience of Emiliania huxleyi to future changes in subantarctic waters
Source: PLoS One. 2023 Nov 2;18(11):e0284415. doi: 10.1371/journal.pone.0284415 (PMC10621989; doi:10.1371/journal.pone.0284415)
Supplement: S4 Fig — (DOCX) [file pone.0284415.s004.docx]

**S4 Fig. Relationship between *E. huxleyi* cell surface area (µm^2^) and A.  PIC (pg cell^-1^) and B. calcification (pg cell^-1^ hr^-1^).**

A

B
