# Supplementary material for: Resilience of Emiliania huxleyi to future changes in subantarctic waters
Source: PLoS One. 2023 Nov 2;18(11):e0284415. doi: 10.1371/journal.pone.0284415 (PMC10621989; doi:10.1371/journal.pone.0284415)
Supplement: S1 Table — (DOCX) [file pone.0284415.s005.docx]

**S5 Table. Calculation to determine decrease in future bulk water PIC content.** From application of one days growth at the N growth rate at D670 (0.631) one cell = 1.88 cells with a PIC of 33.1 pg. At the F growth rate at D670 (0.746) one cell = 2.11 cells after one days growth, with a PIC content of 17.39 ng. The PIC content after one days growth at 2100 is only 52% of the PIC content at 2015.

|  | **Growth rate at D670** | **PIC Content at D670 (ng per cell)** | **Cell number after one day of growth** | **Total PIC content of cells after growth (ng)** | **%age of N PIC content at F conditions** |
| --- | --- | --- | --- | --- | --- |
| **N** | **0.631** | **17.6** | **1.88** | **33.09** |  |
| **F** | **0.746** | **8.24** | **2.11** | **17.39** | **52.5%** |
