# Supplementary material for: Resilience of Emiliania huxleyi to future changes in subantarctic waters
Source: PLoS One. 2023 Nov 2;18(11):e0284415. doi: 10.1371/journal.pone.0284415 (PMC10621989; doi:10.1371/journal.pone.0284415)
Supplement: S1 Appendix — (DOCX) [file pone.0284415.s006.docx]

**S6 Appendix. Regression Analyses.**

**Linear regression results for growth of N population (Fig 1).**

**Linear Regression** Friday, 7 October 2022 3:59:45 pm

**Data source:** Growth over time in Cocco August 2020.JNB

Now = 0.575 + (0.0000774 * Now Time)

N = 69

R = 0.389 Rsqr = 0.151 Adj Rsqr = 0.138

Standard Error of Estimate = 0.041

**Coefficient Std. Error t P**

Constant 0.575 0.00992 57.913 <0.001

Now Time 0.0000774 0.0000224 3.452 <0.001

Analysis of Variance:

**DF SS MS F P**

Regression 1 0.0201 0.0201 11.916 <0.001

Residual 67 0.113 0.00168

Total 68 0.133 0.00195

Normality Test (Shapiro-Wilk) Passed (P = 0.584)

Constant Variance Test (Spearman Rank Correlation): Passed (P = 0.936)

Power of performed test with alpha = 0.050: 0.915

=================

Regression Diagnostics:

=================

**Row Predicted Residual**

1 0.575 -0.0123

2 0.576 -0.0426

3 0.577 0.00200

4 0.578 -0.0989

5 0.578 -0.0991

6 0.579 0.0243

7 0.579 -0.0182

8 0.580 0.00992

9 0.581 -0.0103

10 0.581 0.00339

11 0.582 0.0290

12 0.583 0.0151

13 0.583 0.00807

14 0.585 0.0175

15 0.585 0.00664

16 0.588 0.0402

17 0.589 0.0129

18 0.590 0.00739

19 0.590 0.00245

20 0.591 0.0199

21 0.591 -0.0678

22 0.594 -0.00331

23 0.595 0.0395

24 0.596 0.0146

25 0.597 0.0659

26 0.598 -0.00991

27 0.598 0.0443

28 0.599 0.0957

29 0.600 -0.0409

30 0.600 0.0612

31 0.601 -0.0246

32 0.601 0.0224

33 0.602 0.0236

34 0.604 -0.0580

35 0.607 0.0114

36 0.608 0.0312

37 0.609 -0.0473

38 0.609 0.0550

39 0.610 -0.0538

40 0.611 -0.0594

41 0.612 0.0633

42 0.612 0.0713

43 0.613 0.0596

44 0.614 0.0179

45 0.615 -0.0167

46 0.615 -0.0482

47 0.616 -0.0256

48 0.616 0.0249

49 0.617 -0.00460

50 0.617 0.0298

51 0.618 0.00343

52 0.619 -0.0133

53 0.620 -0.0369

54 0.620 0.0138

55 0.621 -0.0254

56 0.621 0.0145

57 0.622 -0.0870

58 0.624 0.0793

59 0.624 -0.00720

60 0.625 -0.0157

61 0.625 -0.0636

62 0.626 -0.0267

63 0.626 0.00544

64 0.628 0.0235

65 0.628 -0.00350

66 0.629 -0.00603

67 0.630 -0.0149

68 0.630 -0.0325

69 0.631 0.00411

**Linear regression results for growth of F population (Fig 1).**

**Linear Regression** Friday, 7 October 2022 4:03:58 pm

**Data source:** Growth over time in Cocco August 2020.JNB

Future = 0.578 + (0.000244 * Future Time)

N = 65 Missing Observations = 1

R = 0.745 Rsqr = 0.554 Adj Rsqr = 0.547

Standard Error of Estimate = 0.049

**Coefficient Std. Error t P**

Constant 0.578 0.0123 47.176 <0.001

Future Time 0.000244 0.0000276 8.851 <0.001

Analysis of Variance:

**DF SS MS F P**

Regression 1 0.185 0.185 78.348 <0.001

Residual 63 0.149 0.00237

Total 64 0.335 0.00523

Normality Test (Shapiro-Wilk) Passed (P = 0.944)

Constant Variance Test (Spearman Rank Correlation): Passed (P = 0.706)

Power of performed test with alpha = 0.050: 1.000

=================

Regression Diagnostics:

=================

**Row Predicted Residual**

1 0.581 -0.0987

2 0.584 -0.126

3 0.586 -0.0467

4 0.589 0.0519

5 0.591 -0.000275

6 0.595 -0.00894

7 0.598 -0.0120

8 0.599 0.0426

9 0.602 0.0173

10 0.603 0.00886

11 0.606 0.0377

12 0.608 -0.0116

13 0.610 0.0298

14 0.612 0.0401

15 0.621 -0.0548

16 0.623 0.00247

17 0.625 0.0391

18 0.628 0.0170

19 0.631 0.0187

20 0.633 -0.0211

21 0.641 0.0373

22 0.643 -0.0594

23 0.647 0.0296

24 0.650 -0.0178

25 0.652 -0.0364

26 0.653 0.0505

27 0.656 0.0896

28 0.659 0.0255

29 0.661 0.00536

30 0.663 0.0304

31 0.665 -0.0199

32 0.667 0.0336

33 0.682 -0.00714

34 0.684 0.0668

35 0.687 0.106

36 0.691 -0.0597

37 0.694 -0.0914

38 0.695 0.0327

39 0.697 0.0853

40 0.700 0.0485

41 0.702 -0.0114

42 0.704 -0.0371

43 0.706 -0.0692

44 0.708 -0.0741

45 0.710 0.0481

46 0.712 0.00568

47 0.713 0.0595

48 0.717 -0.00142

49 0.718 0.00968

50 0.720 -0.0388

51 0.722 -0.0290

52 0.724 -0.0697

53 0.727 -0.0101

54 0.733 0.0126

55 0.735 -0.0153

56 0.737 -0.0349

57 0.738 -0.0612

58 0.740 -0.0156

59 0.741 0.00520

60 0.747 -0.0430

61 0.748 -0.0625

62 0.750 0.0621

63 0.751 0.0735

64 0.753 -0.0132

65 0.754 0.0355

**Regression results for growth of N and F populations at D720 at different temperatures (Fig 3).**

**NonLinear Regression - Global Curve Fitting Monday, 23 May 2022 9:53:54 am**

**Data Source: Temp Norm May2022 in Cocco.JNB NOW**

**Equation: Peak, Gaussian, 3 Parameter**

f = a*exp(-.5*((x-x0)/b)^2)

**Data Set Specifications:**

**Data Set Independent Variable Dependent Variable**

1 Column 1 Column 2

**Global Parameters:**

a

b

x0

A Global Parameter is shared across all data sets.

**Global Goodness of Fit:**

**R Rsqr Adj Rsqr Standard Error of Estimate**

0.846 0.716 0.695 0.027

**Analysis of Variance:**

**DF SS MS**

Regression 3 4.234 1.411

Residual 26 0.019 7.320E-04

Total 29 4.253 0.147

Corrected for the mean of the observations:

**DF SS MS**

Regression 2 0.048 0.024 Residual 26 0.019 7.320E-04

Total 28 0.067 0.002

**Statistical Tests:**

**Normality Test (Shapiro-Wilk)** Passed (P = 0.0938)

W Statistic= 0.9389 Significance Level = 0.0500

**Constant Variance Test (Spearman Rank Correlation)** Passed (P = 0.1407)

Number of Observations = 29

**Rsqr**  = 0.716

**Residual Sum of Squares** = 0.019

**Parameter Estimates:**

**Coefficient Std. Error t P**

a 0.422 0.008 56.188 <0.0001

b 8.604 0.900 9.559 <0.0001

x0 13.323 0.485 27.486 <0.0001

**Fit Equation Description:**

[Variables]

g_peaksign(q) = if(total(q)>q[1],1,-1)

g_xatymin(q,r) = xatymax(q,max(r)-r)

f0_x = col(1)

f0_y = col(2)

f0_reciprocal_y = 1/abs(f0_y)

f0_reciprocal_ysquare = 1/f0_y^2

f0_reciprocal_x = 1/abs(f0_x)

f0_reciprocal_xsquare = 1/f0_x^2

f0_reciprocal_pred = 1/abs(f0)

f0_reciprocal_predsqr = 1/f0^2

f0_weight_Cauchy = 1/(1+4*(f0_y-f0)^2)

g_x = {f0_x}

g_y = {f0_y}

[Parameters]

s_a = if(g_peaksign(g_y)>0,max(g_y),min(g_y)) ' {{previous: 0.421595}}

s_b = if(fwhm(g_x,abs(g_y))<>0,fwhm(g_x,abs(g_y))/2.2,1) ' {{previous: 8.60387}}

s_x0 = if(g_peaksign(g_y)>0,xatymax(g_x,g_y),g_xatymin(g_x,g_y)) ' {{previous: 13.3231}}

[Equation]

f0 = s_a*exp(-0.5*((f0_x-s_x0)/s_b)^2)

fit f0 to f0_y

[Constraints]

s_b>0

[Options]

tolerance=1e-10

stepsize=1

iterations=200

Number of Iterations Performed = 8

**NonLinear Regression - Global Curve Fitting Monday, 23 May 2022 9:55:01 am**

**Data Source: Temp Norm May2022 in Cocco.JNB FUTURE**

**Equation: Peak, Gaussian, 3 Parameter**

f = a*exp(-.5*((x-x0)/b)^2)

**Data Set Specifications:**

**Data Set Independent Variable Dependent Variable**

1 Column 1 Column 3

**Global Parameters:**

a

b

x0

A Global Parameter is shared across all data sets.

**Global Goodness of Fit:**

**R Rsqr Adj Rsqr Standard Error of Estimate**

0.976 0.952 0.948 0.019

**Analysis of Variance:**

**DF SS MS**

Regression 3 4.950 1.650

Residual 25 0.009 3.577E-04

Total 28 4.959 0.177

Corrected for the mean of the observations:

**DF SS MS**

Regression 2 0.177 0.088 Residual 25 0.009 3.577E-04

Total 27 0.186 0.007

**Statistical Tests:**

**Normality Test (Shapiro-Wilk)** Passed (P = 0.4076)

W Statistic= 0.9629 Significance Level = 0.0500

**Constant Variance Test (Spearman Rank Correlation)** Failed (P = 0.0087)

Number of Observations = 28

**Rsqr**  = 0.952

**Residual Sum of Squares** = 0.009

**Parameter Estimates:**

**Coefficient Std. Error t P**

a 0.496 0.006 89.012 <0.0001

b 7.933 0.464 17.110 <0.0001

x0 14.844 0.406 36.585 <0.0001

**Fit Equation Description:**

[Variables]

g_peaksign(q) = if(total(q)>q[1],1,-1)

g_xatymin(q,r) = xatymax(q,max(r)-r)

f0_x = col(1)

f0_y = col(3)

f0_reciprocal_y = 1/abs(f0_y)

f0_reciprocal_ysquare = 1/f0_y^2

f0_reciprocal_x = 1/abs(f0_x)

f0_reciprocal_xsquare = 1/f0_x^2

f0_reciprocal_pred = 1/abs(f0)

f0_reciprocal_predsqr = 1/f0^2

f0_weight_Cauchy = 1/(1+4*(f0_y-f0)^2)

g_x = {f0_x}

g_y = {f0_y}

[Parameters]

s_a = if(g_peaksign(g_y)>0,max(g_y),min(g_y)) ' {{previous: 0.496268}}

s_b = if(fwhm(g_x,abs(g_y))<>0,fwhm(g_x,abs(g_y))/2.2,1) ' {{previous: 7.93286}}

s_x0 = if(g_peaksign(g_y)>0,xatymax(g_x,g_y),g_xatymin(g_x,g_y)) ' {{previous: 14.8436}}

[Equation]

f0 = s_a*exp(-0.5*((f0_x-s_x0)/s_b)^2)

fit f0 to f0_y

[Constraints]

s_b>0

[Options]

tolerance=1e-10

stepsize=1

iterations=200

Number of Iterations Performed = 9
